# Liquid Atmospheric Pressure Matrix-Assisted Laser Desorption/Ionization Mass Spectrometry Using a Commercial Ion Source and Orbitrap Mass Analyzer

**DOI:** 10.1021/acs.analchem.4c04458

**Published:** 2024-09-27

**Authors:** Bob Challen, Lily R. Adair, Rainer Cramer

**Affiliations:** † Department of Chemistry, University of Reading, Whiteknights, Reading, RG6 6DX, U.K.

## Abstract

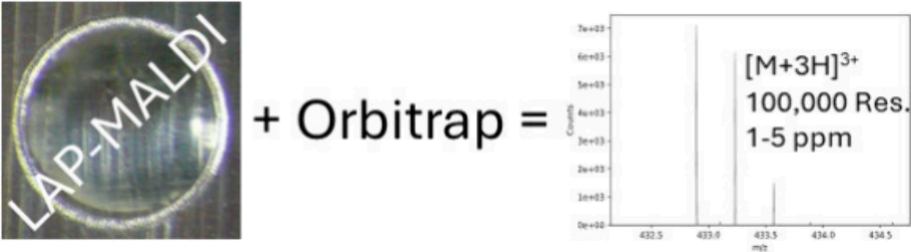

A liquid atmospheric pressure-matrix-assisted laser desorption/ionization (LAP-MALDI) method has been developed and applied to a commercial AP-MALDI source on a hybrid orbitrap mass analyzer. It is shown that electrospray ionization (ESI)-like mass spectra of a range of peptides and proteins can be acquired by LAP-MALDI mass spectrometry (MS) as previously demonstrated on a homemade LAP-MALDI-Q-TOF setup but without the need of any modification to the commercially available MS equipment used. Multiply charged peptide ions were recorded with a resolution of around 100,000 and a mass accuracy of less than 5 ppm. The higher resolution and mass accuracy of the orbitrap analyzer compared with previously employed Q-TOF instrumentation provided high confidence in bacterial proteoform and species identification by top-down protein analysis.

In the past decade, liquid atmospheric pressure-matrix-assisted laser desorption/ionization (LAP-MALDI) has substantially extended the capabilities of soft ionization techniques and thus the possibilities of MS analysis.^1−4^ Although its name indicates a close relation to conventional (solid-state) MALDI, LAP-MALDI is in fact rather a hybrid between MALDI and ESI. However, it is not a hyphenation of soft laser desorption and ESI as can be found in MALDESI,^5^ LAESI,^6^ and ELDI;^7^ neither should it be compared to laserspray or similar laser ionization techniques^8,9^ since LAP-MALDI only ablates minute amounts of sample (<pL) at relatively low laser energies per pulse, thus being far less consuming and more sensitive, and provides a durable and stable ion yield.^1^

Recent analytical advances achieved by LAP-MALDI MS include record-breaking sample analysis speeds of up to 60 samples per second^10^ (10 samples per second for protein analysis^11^) and LAP-MALDI MS profiling analysis to diagnose disease faster and earlier than other current methods as shown by the detection of bovine tuberculosis^12^ and mastitis.^13^

The advantages and new functionalities of LAP-MALDI MS can be explained by the unique combination of using a nanosecond-pulsed laser with a high pulse repetition rate as a precise probe for molecular desorption and the use of truly liquid droplets as samples. The former allows for extremely high sampling rates (roughly only 10× less than the laser pulse repetition rate); highly stable laser pulse energies; and spatially well-defined and, in combination with appropriate liquid matrices, small desorption/ablation volumes, thus maximizing the sample analysis speed while limiting the consumed sample amount. Liquid droplets allow for homogeneous samples, stable ion yields, and sample environments that are far more native when compared to conventional (crystalline) MALDI. Together with a heated inlet capillary, LAP-MALDI also produces predominately ESI-like multiply charged peptides/proteins, allowing the detection of high-mass species on high-performing mass spectrometers with a limited *m*/*z* range.

Previously, we have shown that LAP-MALDI can be used on Q-TOF instrumentation with a mass accuracy of ∼10 ppm and a resolution of ≤20,000. However, in many biological MS applications, such as protein analysis, orbitraps have proven to be the mass analyzers of choice with mass accuracies around 1 ppm and mass resolutions of 100,000 or more.

Here, we report for the first time LAP-MALDI MS analyses of peptides and proteins on an orbitrap mass analyzer. In contrast to our previous work on Q-TOF instrumentation, we have used only commercially available equipment. Therefore, the above-described advantages of the orbitrap and LAP-MALDI, apart from high-speed applications, which require faster sample stages, can now be realized by the entire MS community with minimal effort.

## Experimental Section

### Instrumentation

For LAP-MALDI-orbitrap and conventional AP-MALDI-orbitrap analysis, an LTQ Orbitrap XL (Thermo Fisher Scientific, Bremen, Germany) was used in positive ion FTMS mode with an AP/MALDI (ng) UHR source (MassTech, Columbia, MD, USA). The AP-MALDI source used a solid-state 355 nm Nd:YAG laser, with a repetition rate between 1 and 10 kHz and customizable attenuation. For conventional AP-MALDI analyses, a 1 kHz repetition rate with 90% attenuation was used. For LAP-MALDI analyses, a 10 kHz repetition rate with 80% attenuation was used. For all analyses, the LTQ Orbitrap XL capillary temperature was set to 400 °C, the resolution was set to 100,000, and the max. inject time was set to 1000 ms, resulting in a scan time of approximately 2.8 s/scan. For MS analyses, the *m*/*z* range was set to 200–3000, and for MS/MS analyses, the *m*/*z* range was set to 200–2000. Collision energies were in the range of 35–40 V.

For LAP-MALDI-QTOF analyses, a Synapt G2-Si (Waters, Wilmslow, UK) was used in positive ion sensitivity mode with an in-house-built AP-MALDI source. A detailed description of the in-house AP-MALDI source can be found in earlier publications.^14^ In brief, a stainless-steel target plate was positioned perpendicular to a heated inlet ion transfer tube, consisting of a stainless-steel tube coated with thermal cement and wrapped in a high-resistivity wire. A pulsed 343 nm DPSS laser (Coherent, Santa Clara, CA, USA) with a laser pulse width of approximately 1 ns was used to irradiate the sample at an angle of incidence of 60°. The laser beam was attenuated to produce pulse energies of approximately 10 μJ at a focal diameter of approximately 50–100 μm with a laser pulse repetition rate of 30 Hz. The power supplied to the heated capillary was set to 30 W, and a N_2_ gas counterflow of 180 L/h was applied to the source, resulting in an ion transfer tube temperature of approximately 200–250 °C. The *m*/*z* range of the Synapt G2-Si was set to 50–3000, with the scan time set to 1 s/scan.

### Materials

HPLC-grade isopropanol and LC-MS-grade water, acetonitrile, methanol, and trifluoroacetic acid (TFA) were purchased from Fisher Scientific (Loughborough, UK). Ethylene glycol was purchased from Merck (Gillingham, UK). The eight peptide standards (leucine-enkephalin, angiotensin I, angiotensin II, bradykinin, substance P, ACTH Clip [1–17], and melittin) and three protein standards (ubiquitin from bovine erythrocytes, cytochrome C from equine heart, and myoglobin from equine skeletal muscle) were purchased from Merck (Gillingham, UK). α-Cyano-4-hydroxy-cinnamic acid (CHCA) was purchased from Bruker (Coventry, UK). Dehydrated nutrient agar culture medium was obtained from Oxoid/ThermoFisher (Basingstoke, UK). The *Klebsiella pneumoniae* (NCTC 9633) bacterial strain was obtained as freeze-dried discs from Pro-Lab Diagnostics (Wirral, UK).

### Analyte Preparations

All peptide and protein standards were first individually dissolved in water to create 100 μM stock solutions. For both peptide and protein mixtures, the respective stock solutions were combined and diluted with water to obtain solutions at the 10 μM level for each individual analyte. These and further dilutions with water were used to generate MALDI samples.

For the bacterial analysis, a loopful of *Klebsiella pneumoniae* (NCTC 9633) stock stored in 70% glycerol was cultured on solid nutrient agar medium at 37 °C for 24 h. Approximately 5 μL of biological material was harvested and resuspended in 50 μL of 80% TFA and precipitated for 30 min before the addition of 450 μL of water. The sample was subsequently centrifuged for 10 min at 4,000*g*. The resulting pellet was washed once with 300 μL of water/acetonitrile/isopropanol (1/1/1, v/v/v) before being resuspended in 30 μL of a fresh solution using the same composition.

### MALDI Matrix and Sample Preparations

For conventional (solid) AP-MALDI matrix preparations, water/acetonitrile (1/1, v/v) was added to CHCA to create a solution of 10 mg/mL. This solution was then thoroughly vortexed until a full dissolution was achieved. For LAP-MALDI matrix preparations, water/acetonitrile (7/3, v/v) was added to CHCA to create a solution of 20 mg/mL. This solution was then thoroughly vortexed to achieve full dissolution, at which point ethylene glycol was added using a volume that was 60% of the initial solution, followed by additional vortexing.

To prepare the MALDI samples, the matrix solution was first mixed 1:1 (v:v) with the analyte solution. For conventional AP-MALDI analyses, a dried droplet sample preparation method was employed by spotting 0.5 μL of the matrix/analyte solution and allowing it to dry under ambient conditions. For LAP-MALDI analyses, 0.5 μL of the matrix/analyte solution was spotted onto the target plate and was ready to be analyzed.

### Data Processing

To ensure that the data from the LTQ Orbitrap XL and Synapt G2-Si were processed similarly, the raw data files were first converted into mzML format. The data were then extracted with a custom Python script using the pyOpenMS package.^15^ The Python script is available free of charge and included within the data repository at the University of Reading Research Data Archive. Unless otherwise specified, 30 scans were combined per spectrum.

*De novo* sequencing was achieved by finding sequence tags using the *m*/*z* differences of the major fragment ions. These were then searched using MS-Pattern (ProteinProspector v 6.5.0; https://prospector.ucsf.edu/) against “UniProtKB.2020.09.02” with “Microorganisms” as taxonomy and “0” for the maximum number of mismatched amino acids. The *Klebsiella pneumoniae*-specific proteoform of the most common protein match (major outer membrane lipoprotein) was then further interrogated, and additional y-ions were assigned. The final largest sequence stretch that could be assigned to the fragment y-ions was then blasted in UniProt (https://www.uniprot.org/blast), searching the UniProtKB databases.

## Results and Discussion

All results presented here were obtained within a few days of usage after the commercial AP-MALDI source was installed without any prior experience of operating the AP-MALDI source or the orbitrap mass spectrometer.

**Figure 1:**
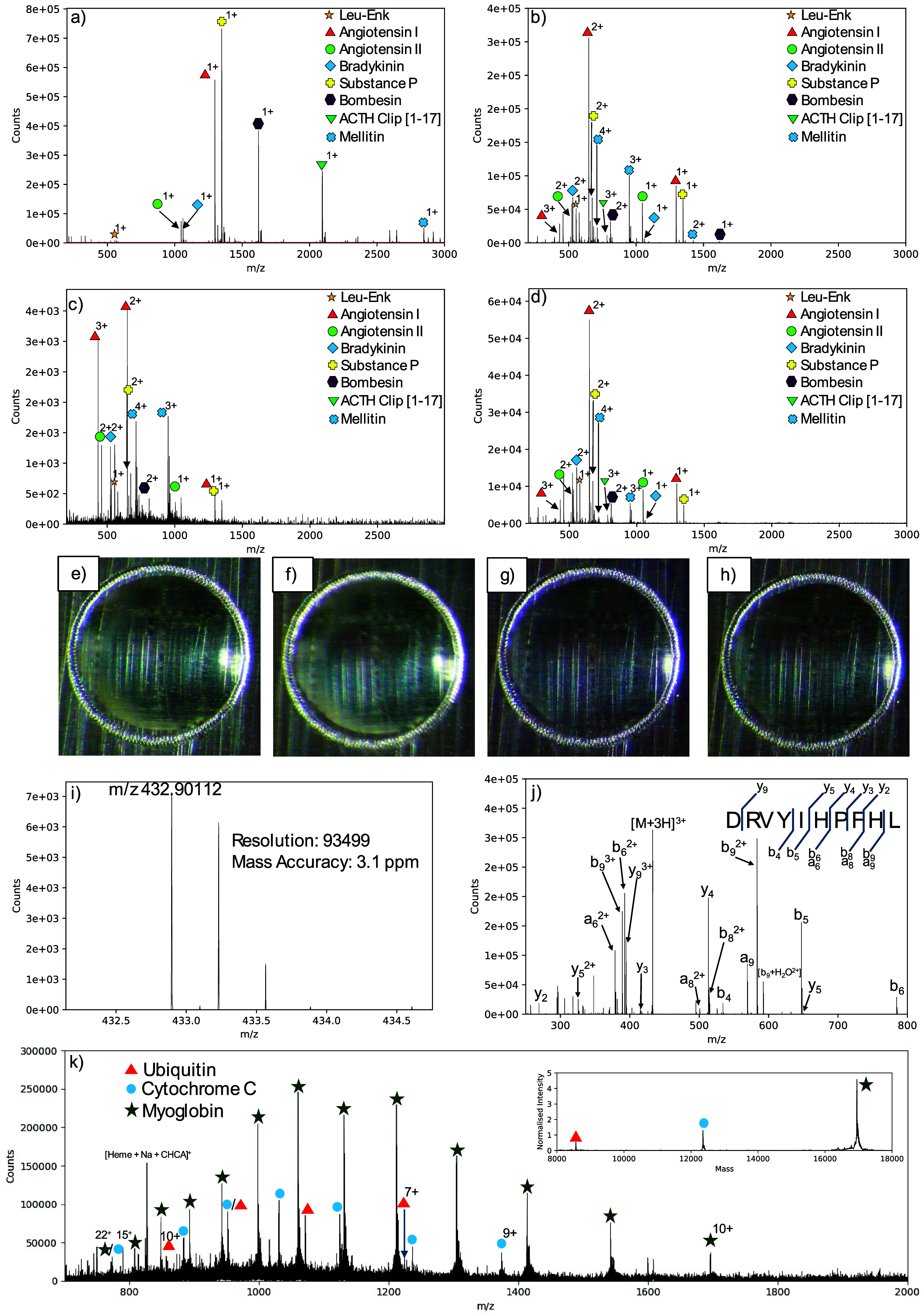
LAP-MALDI-orbitrap analysis of peptides and proteins. (a–d) Single-scan MALDI mass spectra of a peptide mixture using (a) conventional (solid-state) MALDI-orbitrap analysis (dried-droplet solid sample), (b) first LAP-MALDI-orbitrap analysis, (c) LAP-MALDI-Q-TOF analysis using the same sample as for panel b, and (d) second LAP-MALDI-orbitrap analysis using the same sample as for panel b and c. (e–h) Images of the LAP-MALDI sample (droplet diameter: ∼ 2 mm) used for panels b–d: (e) before any analysis, (f) after first LAP-MALDI-orbitrap analysis, (g) after LAP-MALDI-Q-TOF analysis, and (h) after second LAP-MALDI-orbitrap analysis. (i) LAP-MALDI-orbitrap mass spectrum of the triply protonated angiotensin I (50 fmol on target). (j) MS/MS spectrum of the precursor ion shown in panel i. (k) LAP-MALDI-orbitrap mass spectrum of a protein mixture. The inset displays the deconvoluted spectrum.

Figure 1 shows the MS survey scan data obtained for a mixture of eight peptides acquired by solid MALDI (dried-droplet preparation; Figure 1a) and LAP-MALDI (Figure 1b) on an orbitrap mass analyzer with a commercial AP-MALDI source. In comparison, the same liquid sample was also analyzed on our home-built LAP-MALDI source coupled with a Q-TOF instrument by transferring the sample plate between the instruments (Figure 1c) and back to the orbitrap (Figure 1d). Images of this sample clearly show that virtually no changes of the sample droplet are visible, despite the several thousands of laser desorption events that took place at each analysis (Figure 1e–h). Using 50 fmol of angiotensin on target, mass accuracy and resolution for the triply protonated angiotensin I [M+3H]^3+^ (*m*/*z* 432.9) was 3.1 ppm and ∼93,500, respectively (Figure 1i) while the MS/MS data of this peptide ion is typical for a triply protonated angiotensin 1 (Figure 1j). MS data of a protein mixture clearly show the ESI-like charge-state distributions, leading to well-resolved mass peaks in the deconvoluted processed spectrum (Figure 1k).

**Figure 2:**
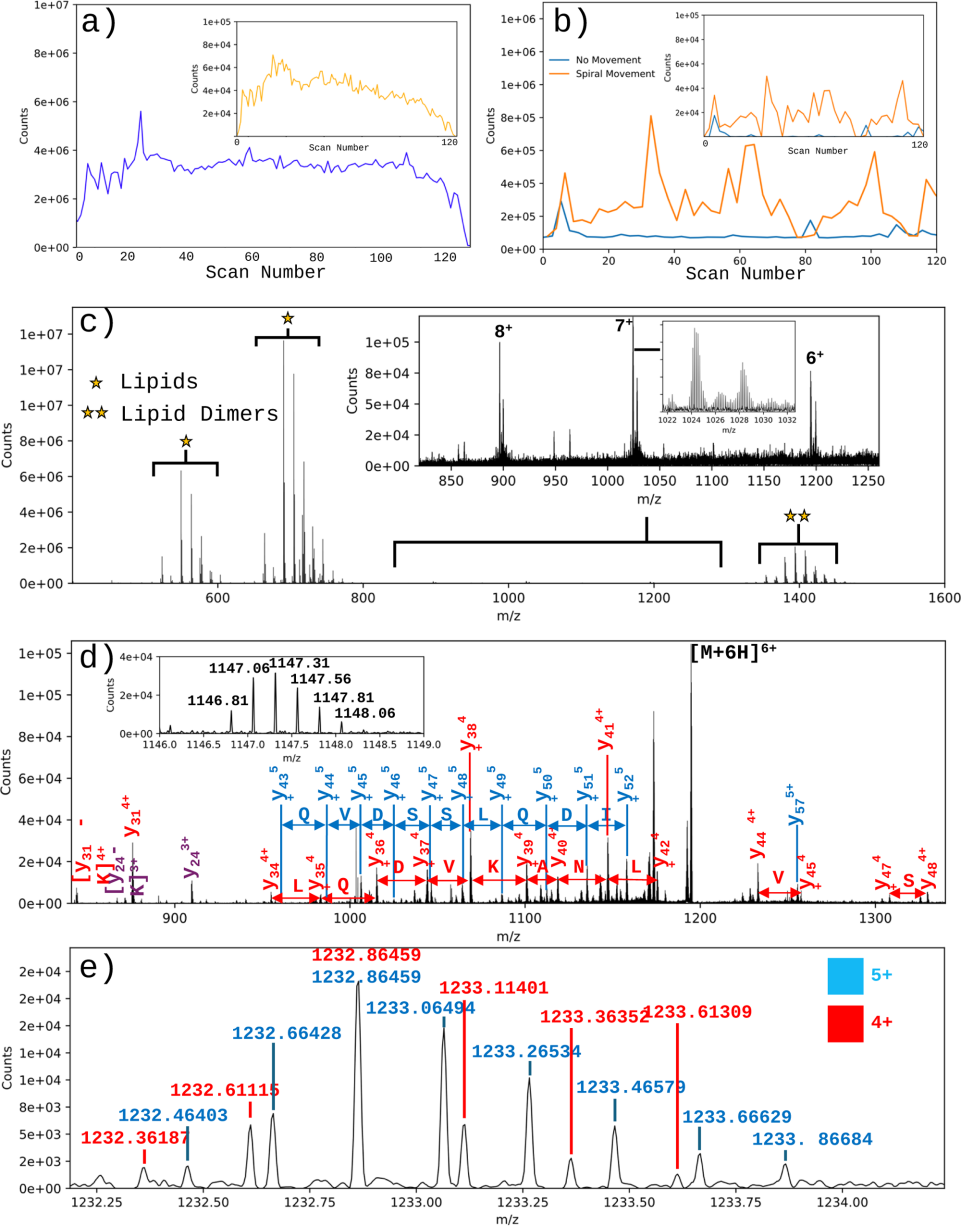
Features and applications of LAP-MALDI-orbitrap MS. (a and b) TIC and EIC (inset) using a peptide mixture for (a) LAP-MALDI-orbitrap analysis (EIC of *m*/*z* 432.9; angiotensin I [M+3H]^3+^) and (b) conventional (solid-state) MALDI-orbitrap analysis (EIC of *m*/*z* 1296.7; angiotensin I [M + H]^+^). (c) LAP-MALDI-orbitrap MS profile of *K. Pneumoniae* extract. (d) MS/MS data of the *m*/*z* 1194 proteoform detected in panel c. Inset shows the y_41_^4+^ fragment ion. (e) Enlarged MS/MS spectrum of panel d, displaying the y_44_^4+^ fragment ion and further strong ion signals of a quintuply charged fragment ion.

The best signal-to-noise ratios were obtained using a maximum laser pulse repetition rate of 10 kHz. Reducing the repetition rate below 3–5 kHz significantly reduced the analyte ion signal, which cannot be attributed to a lower number of desorption events, as the scan numbers were increased accordingly to obtain the same number of laser desorption events per mass spectrum. Thus, this aspect will need further investigation in the future, including synchronization of the pulsed ion beam with downstream ion manipulation/transmission. However, at 10 kHz, current limits of detection for peptides are around 50 fmol (Figure 1i). Ion signal stability is far superior for LAP-MALDI (Figure 2a) compared to conventional (solid-state) MALDI (Figure 2b). Importantly, only a fraction of the LAP-MALDI sample is consumed per laser pulse (<1 millionth) as demonstrated by the duration of the ion signal in both the TIC and EIC (Figure 2a; 2.8 s per scan) over ∼5 min with ∼3 million laser shots (sample ablation events).

In MALDI biotyping, bacterial protein mass profiles mainly consist of ribosomal proteins and are therefore acquired in the *m*/*z* range of up to 12,000 or 20,000, since conventional MALDI predominately produces singly charged peptides/proteins. TOF-only instruments can easily accommodate this *m*/*z*-range requirement, but most high-performance mass spectrometers are not suited to this range. Therefore, having a laser-based soft ionization technique that produces multiply charged peptides/proteins while providing a durable ion yield at good sensitivity can allow for ESI-like analyses with simple, offline sample preparation at high sample-to-sample speed. To demonstrate the advantage of using orbitrap instrumentation for laser-based MS profiling of bacteria, a simple bacterial extract was analyzed using LAP-MALDI. The acquired MS profile (Figure 2c) shows a wealth of species-specific lipids as well as proteoforms. In conventional MALDI-TOF biotyping, using axial TOF instruments, typically both analyte classes are not detected simultaneously, and MS/MS analysis is absent due to its insufficient quality. In this study, the MS/MS data of a bacterial proteoform (Figure 2d), detected in the MS profile of Figure 2c and further analyzed by using the same sample, demonstrate the potential of LAP-MALDI on an orbitrap to provide high-quality sequence information that can result in proteoform identification (Figure 2d) and thus speciation with greater confidence. In this case, a *K. pneumoniae* proteoform of the major outer membrane lipoprotein Lpp was unambiguously identified by *de novo* sequencing. As shown in Figure 2e, top-down proteomic analysis was facilitated by the high resolution of the orbitrap, facilitating the differentiation between overlapping multiply charged fragment ions. The closest UniProt database entry (in June 2024) was A0A0H3GU43, although only the C-terminal y-ion series provided good matches, while neither b-ions nor a match to the precursor ion mass could be found.

## Conclusions

In summary, we demonstrated the implementation of LAP-MALDI on an orbitrap without any further instrument modifications. LAP-MALDI sample preparation is easy and leads to a durable and stable ESI-like ion yield at low sample consumption while providing a high detection sensitivity. The flexible environment and liquid nature of its sample droplet also allow for additional analyses such as native MS^16^ and real-time reaction monitoring, directly from the sample in the ion source. The above-described implementation was instantaneous, required no designated laser(-controlled) area, and used a commercial setup that is already present in many MS laboratories that employ AP-MALDI.

## Data Availability

Data supporting the results reported in this paper are openly available from the University of Reading Research Data Archive at https://doi.org/10.17864/1947.001326
